# Giant chronic calcified subdural empyema: a case report

**DOI:** 10.1186/s13256-020-02429-2

**Published:** 2020-07-11

**Authors:** Amal Saleh Nour, Kibruyisfaw Zewdie Shumbash

**Affiliations:** 1 Department of Radiology, College of Health Sciences, Addis Ababa University, Addis Ababa, Ethiopia; 2 Department of Surgery, College of Health Sciences, Addis Ababa University, Addis Ababa, Ethiopia

**Keywords:** Giant calcified chronic subdural empyema, Computed tomography, Craniotomy

## Abstract

**Background:**

Chronic calcified subdural empyema is an exceedingly rare central nervous system infection with a handful of cases published to date. Reported cases presented with nonspecific clinical signs and symptoms. The duration between the initial onset of symptoms and diagnosis can vary drastically from a few years to up to 46 years. Although there are known predisposing causes, the initial source of infection can sometimes be difficult to identify.

**Case presentation:**

Our patient was a 39-year-old Ethiopian man who presented with left-side body weakness of 6 years’ duration with worsening of symptoms of 6 months’ duration. He had no history of trauma, meningitis, or previous surgery. The results of routine laboratory tests were normal. The diagnosis was made by computed tomography and magnetic resonance imaging and was confirmed by surgery. Frontoparietal craniotomy was performed, and evacuation of non-foul-smelling collection was done. The patient was reoperated for tension pneumocephalus 48 hours after the initial surgery. He died 10 days later.

**Conclusion:**

This is a rare case of a giant chronic calcified subdural empyema with no known preceding history of trauma or infection.

## Introduction

Intracranial subdural empyema is an infection that is contained within the space between the dura and arachnoid mater [1]. Chronic intracranial subdural empyema is a rare central nervous system infection with non-specific clinical manifestations [2, 3]. Imaging with computed tomography (CT) and magnetic resonance imaging (MRI) shows subdural lesions with calcification in the brain as common features [2]. Reviewed reports have stated that cases of partially calcified empyema have become symptomatic 13 years after the initial onset of infection [4]. Diagnosis of chronic subdural empyema has also been made almost 46 years after the onset of symptoms, and the result of histologic examination of the collection can be sterile [5]. We present a case of a patient with a giant chronic calcified subdural empyema presenting 6 years after the initial symptoms. This is an exceedingly rare case with no identifiable predisposing condition that led to the development of chronic calcified subdural empyema. There are only a few case reports with nonspecific clinical presentation, making the diagnosis difficult.

## Case presentation

Our patient was a 39-year-old Ethiopian man who presented with left-side body weakness of 6 years’ duration. He was admitted to our hospital with worsening of symptoms of 6 months’ duration with failure to communicate of 1 week and frequent falling episodes while attempting to walk. His left-side body weakness initially started from the left lower extremity, followed by the left upper extremity. The patient also had worsening of headache, abnormal body movement, and left-side facial deviation, as well as choking episodes. He also complained of occasional urinary incontinence; otherwise, he had no history of trauma, ear discharge, past history of sinusitis, or meningitis treatment. He had no history of fever or previous treatment for tuberculosis. He had no history of smoking or alcohol intake and no history of known medical illness or drug intake. He was a priest working in a church for the past 25 years. He is a father of two and had discontinued his education at grade 6.

His physical examination revealed vital signs of blood pressure 110/80 mmHg, pulse rate 88 beats/minute, respiration rate 16 breaths/minute, and temperature 36.5 °C. A pertinent finding was that upon central nervous system examination, the patient was oriented to time, place, and person. His Glasgow Coma Scale (GCS) score was 15/15. He had left-side supranuclear facial palsy. His muscle power was 4/5 in the left upper and lower extremities. His meningeal signs were negative. Laboratory investigations showed a white blood cell count of 4.91 x 10^3^/μl with 61% neutrophils and 28% lymphocytes and hemoglobin 17.1 g/dl. The patient’s coagulation profile, organ function test, and serum electrolytes were within normal ranges. We did not do purified protein derivative skin test, because the patient had a bacillus Calmette-Guérin vaccination history, but his erythrocyte sedimentation rate was 04 mm/hour. His chest x-ray was normal. He had no history of trauma, meningitis, or previous surgery. The routine laboratory tests were all within the normal range.

**Fig. 1:**
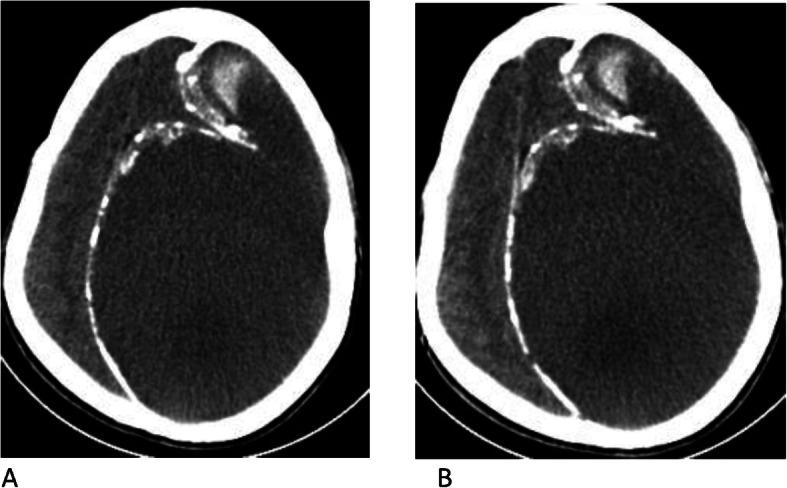
Pre- and postcontrast computed tomographic images. **a** Axial precontrast image showing left frontal, parietal, and temporal convexity calcified extra-axial subdural lesion with hypodensity and anterior focal areas of hyperdensity. **b** Axial postcontrast image showing no enhancement of the lesion

On precontrast brain CT images, there was a left frontal, parietal, and temporal convexity, peripherally calcified, extra-axial, subdural, hypodense collection with focal areas of hyperdensity. There was a mass effect on the adjacent brain parenchyma (Fig. 1a).

Post-contrast images showed no abnormal enhancement of the lesion (Fig. 1b).

**Fig. 2:**
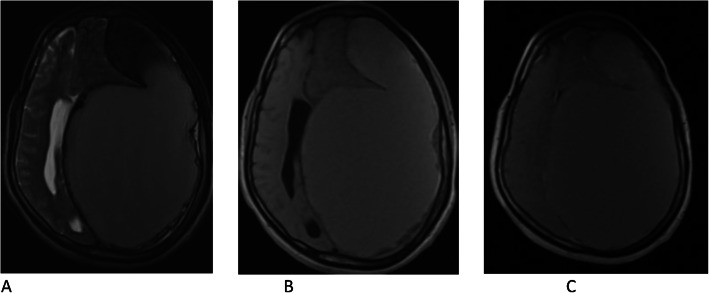
Pre- and postcontrast magnetic resonance imaging. **a** Axial T2-weighted image showing left frontoparietal convexity mixed intensity subdural collection with mass effect on the lateral ventricles. **b** Axial T1-weighted image showing slightly hyperintense signal involving the same location. **c** Axial T1-weighted postcontrast images showing no abnormal contrast enhancement

Brain MRI showed left frontoparietal convexity subdural collection that had mixed iso- and hypointense on axial T2-weighted images and slightly hyperintense signal on axial T1-weighted images with mass effect on the ipsilateral lateral ventricle (Fig. 2a and b). Axial T1-weighted postcontrast images showed no abnormal contrast enhancement (Fig. 2c).

In the preoperative period, the patient was administered analgesic diclofenac 75 mg intramuscularly as needed and deep vein thrombosis prophylaxis heparin 7500 IU subcutaneously twice daily. He took antibiotic ceftriaxone 1 g upon call to the operating room and for the next 24 hours for surgical wound prophylaxis, which was continued 24 hours postoperatively.

Intraoperatively, the pus was sent in three separate tubes: one for cell count, another for chemistry (glucose and protein), and the last one for Gram stain culture (which includes acid-fast bacillus stain and fungal culture), and all of the results were not revealing.

**Fig. 3:**
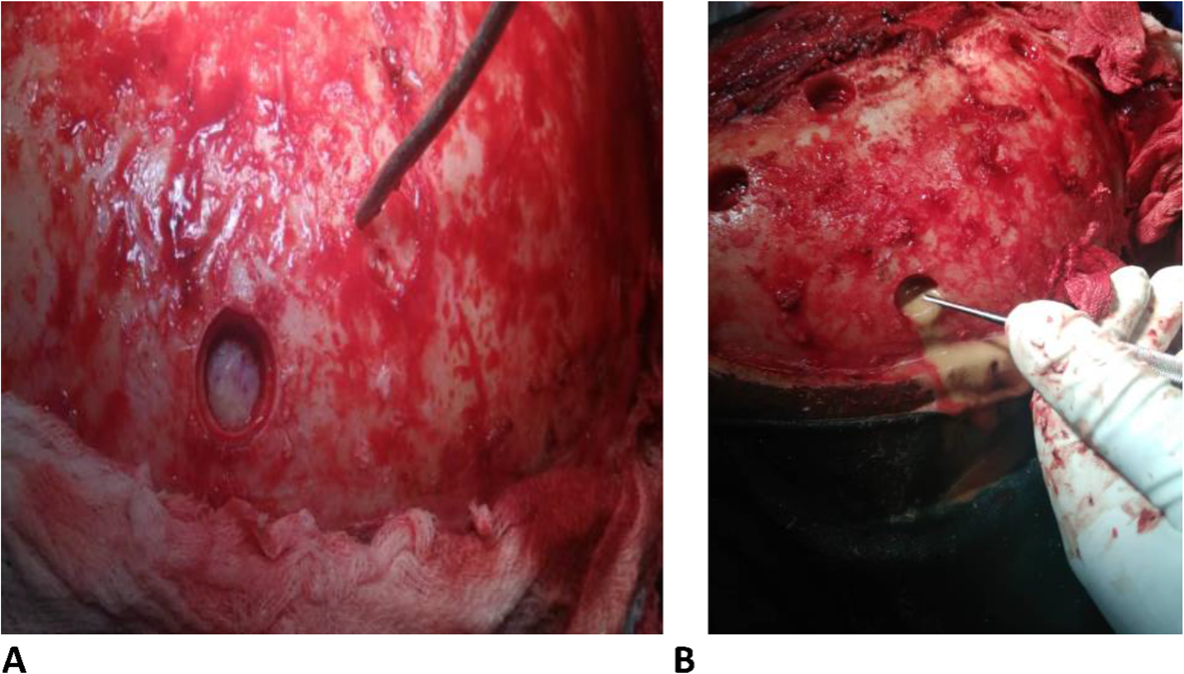
Intraoperative pictures showing the intact dura after a burr hole was made (**a**) and pus pouring through the burr hole opening (**b**)

**Fig. 4:**
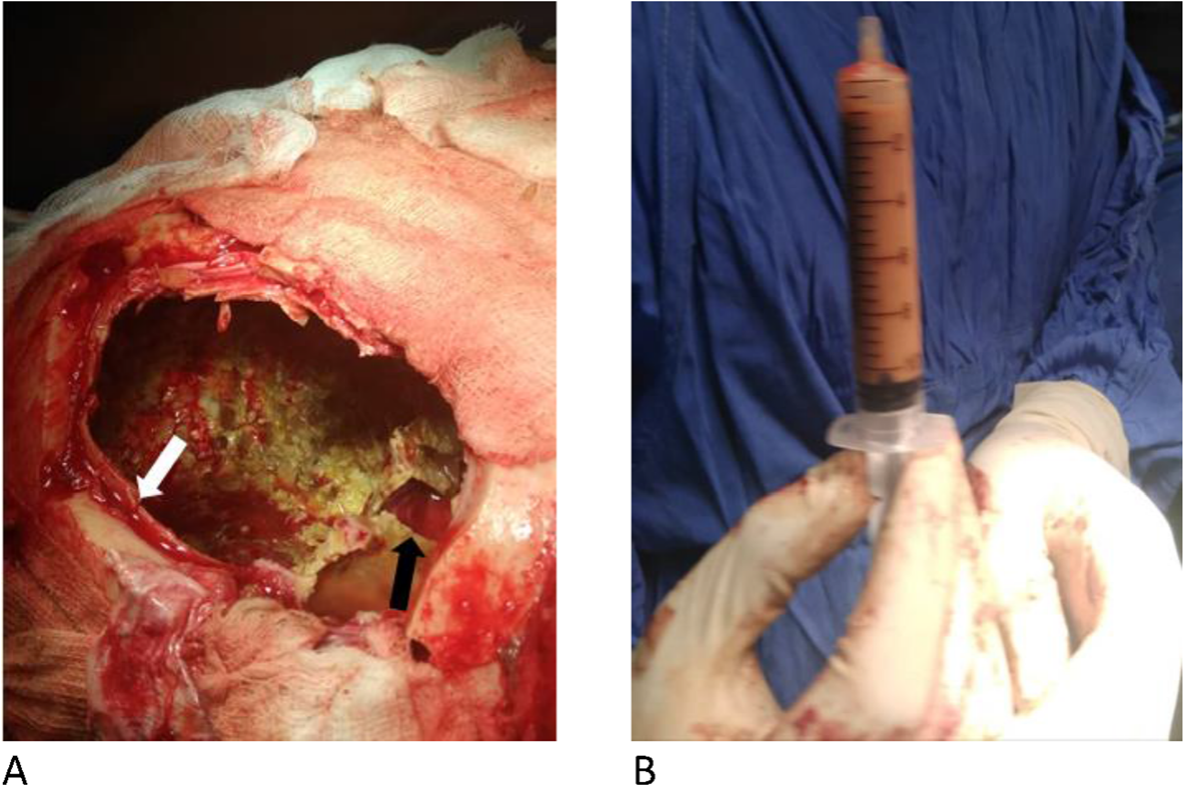
Intraoperative pictures after excision of the outer capsule, indicated with *white arrow*, and biopsy taken from the inner capsule as shown by the *black arrow* (**a**). Evacuated subdural pus (**b**)

The patient underwent left frontoparietal craniotomy in April 2019. Intraoperative findings were a calcified subdural empyema, which was completely covered by the dura. When the outer capsule was resected, there was thick, nonoffensive pus more organized in the frontal area. The pus was evacuated, and the cavity was irrigated with copious amounts of normal saline (Figs. 3 and 4). The inner capsule was not densely calcified compared with the outer capsule, but it was adherent to the underlying pia and cortical vessel. A biopsy was taken, and resection of the inner capsule was deferred. The patient was transferred to the intensive care unit (ICU) and was extubated with a GCS score of 15/15.

**Fig. 5:**
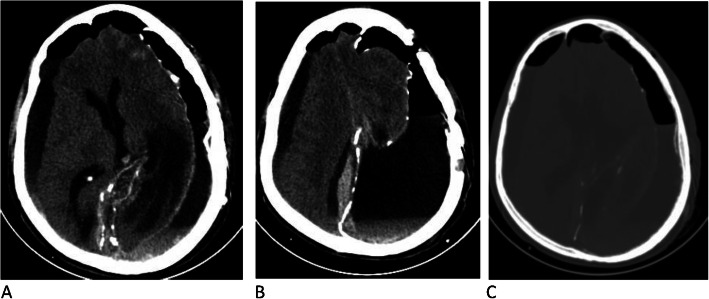
Noncontrast computed tomographic images obtained 48 hours after surgery. Brain window shows bilateral frontoparietal convexity air–fluid level with left parietal hypodensity and mass effect on the left posterior horn of the lateral ventricle (**a** and **b**). Bone window shows the bilateral pneumocephalus (**c**)

Two days after surgery, the patient’s mentation started to deteriorate, and he had two episodes of generalized tonic-clonic seizure. Control CT showed bilateral tension pneumocephalus with subdural collection (Fig. 5a and b). The patient underwent emergency reoperation, and exploration was done. Intraoperatively, there was moderate blood in a mixed subdural collection and a gush of air came out upon elevation of the bone. The patient was transferred to the ICU and intubated and was started on ceftazidime 2 g intravenously three times daily and vancomycin 1 g intravenously twice daily. During his stay in the ICU, blood, urine, and cerebrospinal fluid cultures were performed. The initial culture as well as the subsequent cultures for all three samples did not show any growth through the 14th day. Despite the high dose of broad-spectrum antibiotic, the patient continued to have persistent high-grade fever, and subsequently he developed shock with multiorgan failure. The result of the culture from the evacuated pus was negative. Our patient died 10 days after surgery with a possible diagnosis of aseptic meningitis. An autopsy was not performed.

## Discussion

This is a case report of a patient who presented with a 6-year history of progressive body weakness, and a diagnosis of giant left-side chronic calcified subdural empyema was made. The case is unusual in that there is no identifiable predisposing condition that led to the development of the empyema.

Intracranial subdural empyema is an infection that is contained within the space between the dura and arachnoid mater [1]. Intradural subdural empyema constitutes 15% to 25% of pyogenic intracranial infections and is more common in males. Meningitis is said to be the most common cause of subdural empyema in infants, whereas paranasal infection and otitis media are the most common sources of infection in adults [6]. Other causes include penetrating head trauma, neurosurgical procedure, and seeding from distant sites of infection such as endocarditis. The pathogenesis of subdural empyema results from subdural effusion with late secondary infection due to subclinical episodes of meningitis that ultimately results in the spread of infection into the subdural space, leading to subdural empyema [7]. Chronic calcified subdural empyema is a rare central nervous system infection with nonspecific clinical manifestations. In a review of four cases, patients did not usually present with fever or increase in white blood cell count, which may be due to the localization of the empyema and calcification of the tissues surrounding the lesions [2]. The causes of the infection were also uncertain in other case reports [2].

Although some reported cases have been able to grow bacteria on culture, the pus in our patient’s case was sterile on culture. This has also been reported in another case in which there was an identifiable focus of infection, but the pus was sterile on culture in some of the cases [3]. The dura was adherent in our patient, but this has also been reported in another case in which the dura was very tight and adherent to the underlying capsule.

Case reports compose the main literature available regarding the description of chronic calcified subdural empyema. Surgical removal of empyema is believed to have a medium long-term outcome with surgical treatment [2]. Although the advised treatment is surgery with good outcomes of patients in the mentioned case reports, our patient died 10 days following intervention. There was no identifiable source of infection for the uncontrolled high-grade fever with no significant response to first-line antibiotics.

## Conclusion

This report describes an infrequently encountered case of giant chronic calcified subdural empyema in which the patient had no identifiable preexisting conditions that predisposed him to develop such a large calcified subdural collection. Although the recommended treatment option is surgery, our patient’s cause of death was not established.
